# Defining the inflammatory signature of human lung explant tissue in the presence and absence of glucocorticoid

**DOI:** 10.12688/f1000research.10961.1

**Published:** 2017-04-11

**Authors:** Tracy L Rimington, Emily Hodge, Charlotte K Billington, Sangita Bhaker, Binaya K C, Iain Kilty, Scott Jelinsky, Ian P Hall, Ian Sayers

**Affiliations:** 1 Division of Respiratory Medicine, University of Nottingham, Nottingham, UK; 2 Department of Mechanical Engineering, Kathmandu University, Dhulikhel, Nepal; 3 Inflammation & Remodelling Research Unit, Pfizer Inc, Cambridge, MA, USA

**Keywords:** COPD, asthma, chemokines, inflammation, lung, multiplex, luminex, tissue explant, ex-vivo

## Abstract

Background: Airway inflammation is a feature of many respiratory diseases and there is a need for newer, more effective anti-inflammatory compounds. The aim of this study was to develop an *ex vivo* human lung explant model which can be used to help study the mechanisms underlying inflammatory responses and which can provide a tool to aid drug discovery for inflammatory respiratory diseases such as asthma and COPD.

Method: Parenchymal lung tissue from 6 individual donors was dissected and cultured with two pro-inflammatory stimuli, lipopolysaccharide (LPS) (1 µg/ml) and interleukin-1 beta (IL-1β) (10 ng/ml) in the presence or absence of dexamethasone (1 µM).  Inflammatory responses were assessed using Luminex analysis of tissue culture supernatants to measure levels of 21 chemokines, growth factors and cytokines.

Results: A robust and reproducible inflammatory signal was detected across all donors for 12 of the analytes measured following LPS stimulation with a modest fold increase (<2-fold) in levels of CCL22, IL-4, and IL-2; increases of 2-4-fold in levels of CXCL8, VEGF and IL-6 and increases >4-fold in CCL3, CCL4, GM-CSF, IL-10, TNF-α and IL-1β.  The inflammatory signal induced by IL-1β stimulation was less than that observed with LPS but resulted in elevated levels of 7 analytes (CXCL8, CCL3, CCL4, GM-CSF, IL-6, IL-10 and TNF-α).  The inflammatory responses induced by both stimulations was supressed by dexamethasone for the majority of analytes.

Conclusions: These data provide proof of concept that this *ex vivo* human lung explant model is responsive to inflammatory signals and could be used to investigate the anti-inflammatory effects of existing and novel compounds.  In addition this model could be used to help define the mechanisms and pathways involved in development of inflammatory airway disease.

Abbreviations: COPD: Chronic Obstructive Pulmonary Disease; ICS: inhaled corticosteroids; LPS: lipopolysaccharide; IL-1β: interleukin-1 beta; PSF: penicillin, streptomycin and fungizone

## Introduction

Obstructive lung diseases such as asthma and Chronic Obstructive Pulmonary Disease (COPD) are characterised by inflammation which can affect both large and small airways ^1^. Treatment options for these inflammatory lung diseases remain limited and not all patients respond to the most commonly used medicines, including inhaled corticosteroids (ICS) and β-2 adrenergic receptor agonists ^2– 5^. There is a need for new treatments for both asthma and COPD, and particularly for approaches which target inflammation ^4^, especially in the small airways, which have been increasingly recognised as an important site of inflammation ^6, 7^.

Whilst there have been some studies which have used *ex vivo* cell or tissue to look at inflammatory responses, the lack of a robust human tissue system has to some extent hindered pre-clinical drug development and mechanistic studies in these diseases. Animal models have long been used to try and predict efficacy in human disease but findings in animal models often fail to predict responses in humans. This is particularly true for diseases such as asthma and COPD for which animal models are only able to recapitulate some of the features of the human disease ^2, 8, 9^. A human tissue explant model would therefore complement those *in vivo* mouse models which currently exist.

Preliminary data exist demonstrating that *ex vivo* human lung tissue models can be used to study the effect of allergens and other inflammatory stimuli on selected cytokine responses ^10, 11^. Our aim was to develop a reproducible human lung tissue explant model which could be used for target validation and to help investigate mechanisms underlying inflammation relevant to airway disease.

In this study we assessed human lung tissue explants *ex vivo* to define inflammatory signalling using multiplex cytokine assays. In order to elicit an inflammatory response in human lung tissue bacterial lipopolysaccharide (LPS) and interleukin-1 beta (IL-1β) were used ^12– 14^. We defined the cytokine and chemokine signature of this tissue in response to these stimuli and also provide data on the reproducibility of this model by assessing responses to 21 chemokines, growth factors and cytokines. To determine the usefulness of the model to identify anti-inflammatory mechanisms we also examined the effect of potential inhibitory responses using dexamethasone.

## Methods

Human parenchymal lung tissue was obtained from the Nottingham Research Biorepository from patients undergoing lung resection surgery at Nottingham University Hospitals, UK. Written consent was obtained from all patients and the study was approved by North West 7 REC – Greater Manchester Central (ethics reference 10/H1008/72). The patient demographics of the six donor subjects used in the current study are shown in Supplementary table 1. The mean age of donors was 75.5 ± 10.5 years (4 females and 2 males). In total, three individuals were ex-smokers (stopped ≥ 3 years), two were recent smokers (stopped ≤ 3 years) and one was never a smoker. Three subjects had spirometry suggesting the presence of COPD.

Lung tissue was dissected into 30–100 mg (wet weight) pieces and incubated for 24h in RPMI 1640 (with 2.05 mM L-glutamine and 25 mM HEPES) (Sigma, 51536C) containing Antibiotic Antimycotic Solution (PSF, penicillin, streptomycin and fungizone) (Sigma, A5955). Following initial incubation, media was replaced, and following the addition of LPS or IL-1β (1 µg/ml or 10 ng/ml respectively) or vehicle controls in the presence or absence of 1 µM dexamethasone, the tissue was incubated for a further 24h, followed by the collection of supernatants. All experimental conditions were prepared in duplicate.

We designed a custom multiplex panel of 21 Luminex assays to provide comprehensive information on the protein secretory profile of the human lung tissue. This panel was designed to encompass the main inflammatory pathways activated in the lung, including chemokine, cytokine and growth factor pathways ( Supplementary table 2).

Luminex assays (supplied by R&D, product code LXSAHM) were performed according to the manufacturer’s recommendations using a custom Magnetic Luminex Screening Assay with a Human Premixed Multi-Analyte Kit (R&D systems). Each duplicate supernatant from the lung tissue explant experiment was assayed in duplicate.

Results were normalised using wet tissue weights in individual experiments and data were normalised to maximal inflammatory stimulus level (i.e. LPS or IL-1β, 100%) in each experiment prior to combining data. Statistical analysis was performed using ANOVA and post-hoc Dunnett’s multiple comparisons test. Statistical analysis was performed using GraphPad Prism software (Version 6, GraphPad Software Inc.).

## Ethics approval and consent statement

Written consent was obtained from all patients and the study was approved under ethics reference 10/H1008/72, 12/SC/0526 and 08/H0304/56+5. All samples were obtained and research conducted under the approval of the Nottingham Health Science Biobank, Arden Tissue Bank and Papworth Hospital Research Tissue Bank. Written consent was obtained from all patients to publish research findings obtained from the use of patient samples under the approval of the Research Tissue Banks.

## Results

**Figure 1.**
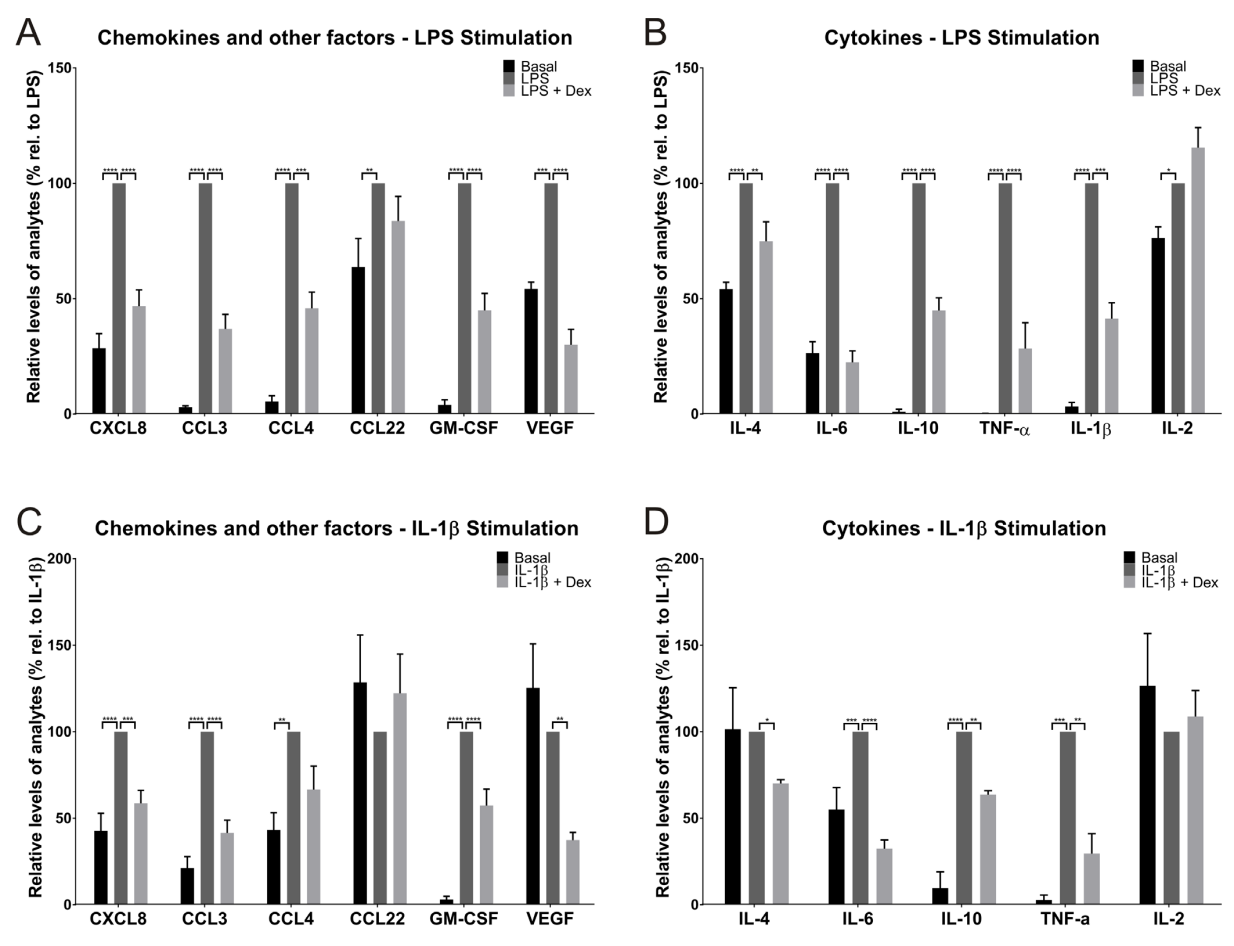
The secretory profile of *ex vivo* human lung tissue following LPS and IL-1β stimulation. LPS significantly induced the release of 12 analytes in cultured lung tissue explants, including chemokines and other factors (e.g. growth factors) ( **A**) and cytokines ( **B**). For 10 of these analytes, this response was attenuated with dexamethasone treatment. Results were normalised using tissue mass and data were then normalised to the LPS stimulation (100%) from each donor and are presented as mean (±SEM, n=6). IL-1β significantly induced the release of 7 analytes in cultured lung tissue explants, including chemokines and other factors ( **C**) and cytokines ( **D**). For 6 of these analytes, this response was attenuated with dexamethasone treatment. Results were normalised using wet tissue mass and data were then normalised to the IL-1β stimulation (100%) from each donor and are presented as mean (±SEM, n=4). Due to limited tissue availability for two donors, it was not possible to obtain tissue from all six donors for the IL-1β experiments.

Using the multiplex approach, 12 of the 21 inflammatory analytes assayed generated quantifiable signals in the Luminex assay across all donors following 24h incubation under baseline (unstimulated) conditions ( Figure 1 and Supplementary table 3). The analytes detected were a range of chemokines including CCL3, CCL4, cytokines including IL-6, CXCL8 and several growth factors including VEGF ( Figure 1 and Supplementary table 3).

With the exception of IL-2, there was a significant induction of levels of all analytes detectable following LPS stimulation ( Figure 1A and 1B). The fold stimulation within donor samples was reasonably reproducible for CXCL8 (3.5-fold), CCL3 (~33-fold), CCL4 (~18-fold), CCL22 (1.6-fold), GM-CSF (~25-fold) and VEGF (1.8-fold) ( Figure 1A). There was also a significant cytokine induction in the tissue, characterised by elevated levels of IL-4 (1.8-fold), IL-6 (3.8-fold), IL-10 (~96-fold), TNF-α (~600-fold), IL-1β (~30-fold) and IL-2 (1.3) ( Figure 1B). The absolute values (as opposed to the fold stimulations) varied to some extent across donors even when corrected for tissue wet weight.

From the 12 analytes that exhibited a significant LPS driven response, pre-treatment with dexamethasone (1 µM) attenuated this response by >50% for 9 of the analytes. Dexamethasone was unable to significantly attenuate the stimulation of CCL22 or IL-2 production, suggesting this induction was steroid insensitive ( Figure 1A and 1B).

IL-1β was also able to induce an inflammatory response in the human lung tissue, however both the magnitude and diversity of the responses observed across the 21 analytes was diminished in comparison to LPS. IL-1β stimulated production of 7 of the analytes, and this response was attenuated by dexamethasone treatment for 6 of these targets ( Figure 1C and 1D). The greatest level of induction was observed for TNF-α (~35-fold), followed by GM-CSF (~32-fold), IL-10 (~10-fold), CCL3 (~5-fold), CCL4 (2.3-fold), CXCL8 (2.3-fold) and IL-6 (1.8-fold) ( Figure 1C and 1D). Treatment with dexamethasone attenuated these inflammatory responses to varying degrees, with the greatest reduction being TNF-α (~70%), although the actual concentration of this analyte was low (~0.8 pg/ml/mg tissue) compared to the LPS stimulated sample (~30 pg/ml/mg tissue) ( Supplementary table 3). Attenuation of the inflammatory response was >35% for the remaining 5 analytes, for which statistically significant reductions were seen for IL-6, CCL3, GM-CSF, CXCL8 and IL-10. Although there was a 33% reduction in CCL4 following treatment with dexamethasone, this was not statistically significant ( Figure 1C and 1D).

**Figure 2.**
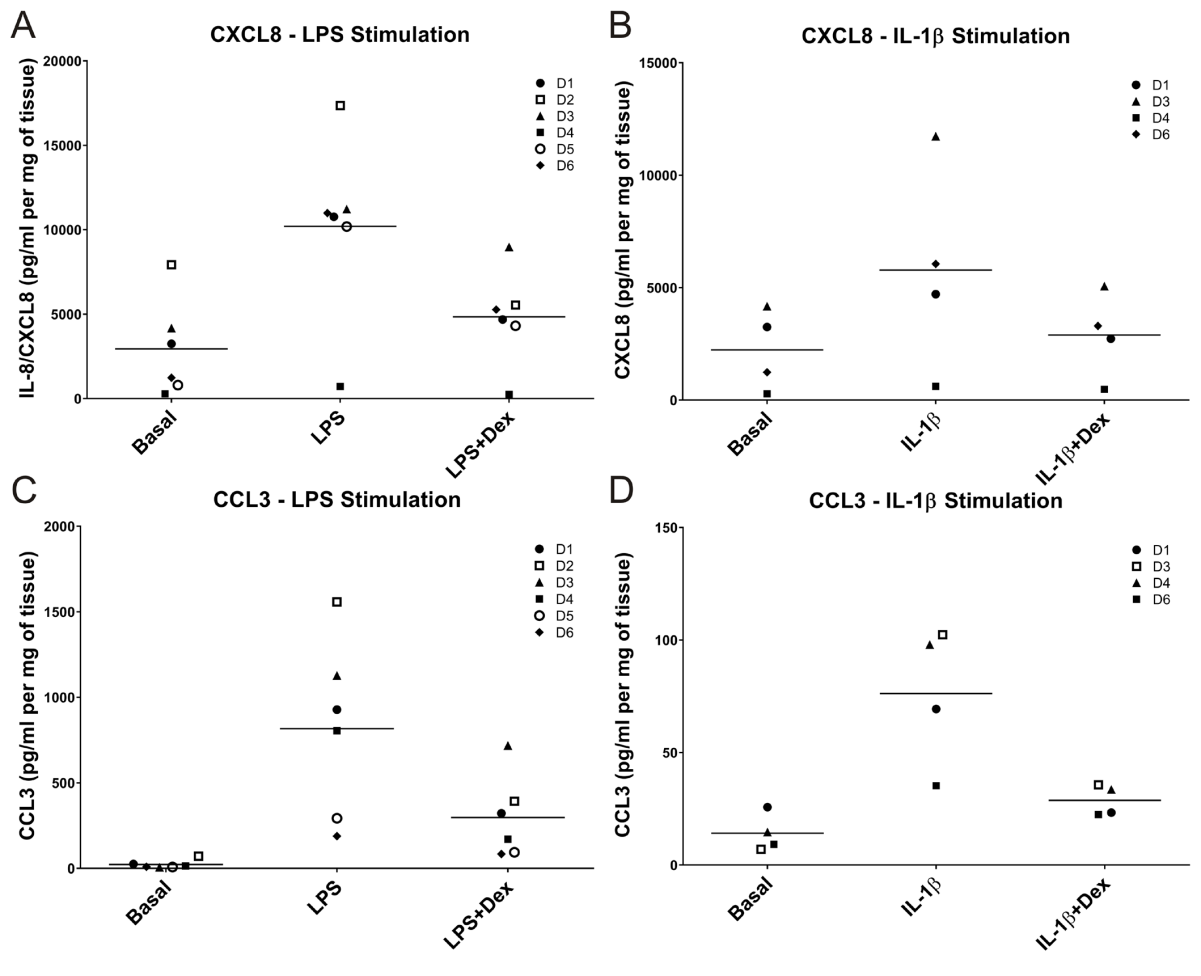
Comparison of concentration of CXCL8 and CCL3 following LPS and IL-1β stimulation and dexamethasone treatment LPS and IL-1β both induced an inflammatory response in *ex-vivo* lung tissue, although the response with LPS was greater than with IL-1β. Compared to the IL-1β stimulation, there was an overall 1.8-fold increase in CXCL8 concentration ( **A** and **B**) following LPS stimulation and an overall 11-fold increase in levels of CCL3 ( **C** and **D**).

LPS induced a more pronounced inflammatory response than IL-1β when comparing absolute concentrations of the analytes measured ( Supplementary table 3). Figure 2 allows direct comparisons to be made between the two pro-inflammatory stimuli, and provides insight into the degree of inter-donor variability observed in the model. Some donor variation was apparent for both CXCL8 and CCL3 at both basal levels and following stimulation ( Figure 2A–D).

In order to further explore the degree of variability in responses, we measured CXCL8 production in tissue obtained from 5 additional subjects. The mean basal levels of CXCL8 produced (total of n=11 donors) were 1941 (range 232–7927) pg/ml/mg tissue and the fold stimulation observed with LPS was 3.9-fold (range 2.2–12.7).

## Discussion

There is a need for well-characterised human lung tissue models to assess pro and anti-inflammatory responses in the lung and to help with target validation during the drug development process. We have therefore developed an explant model using *ex-vivo* human lung tissue to investigate the inflammatory responses induced using two physiologically relevant stimuli. We characterised responses using Luminex assays to simultaneously permit analysis of a range of cytokines and other mediators. We chose LPS as a stimulus to mimic bacterial infection and IL-1β as a more selective pro-inflammatory signal. The data presented demonstrate that reasonably reproducible responses can be obtained in this model despite there being an inevitable element of heterogeneity in the tissue obtained from each donor. We also used pre-treatment with dexamethasone as proof of concept to identify anti-inflammatory effects in this model. The reduction in inflammatory mediator responses observed after dexamethasone pre-treatment support the use of this model for investigation of potentially anti-inflammatory effects of novel compounds in the human lung.

Models currently used in airway disease research have limitations. For instance, rodent *in vivo* models have been heavily relied upon and whilst these can provide useful mechanistic insights they do not always translate well when assessing efficacy in human disease ^8, 9^. Human tissue based models should enhance mechanistic and pre-clinical studies and will hopefully prove more predictive for target validation for diseases such as asthma and COPD.

We describe here the inflammatory secretory profiles obtained using pro-inflammatory stimuli LPS and IL-1β in this model. Both induced release of a range chemokines, cytokines and growth factors. As would be expected, the magnitude of effect was greater with LPS than with IL-1β. Appropriate vehicle controls were included in all experiments and did not produce responses. Some variability in both basal and stimulated levels of mediators was seen between donors, although within donor reproducibility of responses was generally good ( Figure 2).

Bacterial infection and exacerbation are common in COPD and asthma patients ^12, 13, 15– 17^. The broad secretory profile that is obtained following LPS stimulation supports its role as a broad activator of intra-cellular signalling pathways. The responses we observed in the *ex vivo* model broadly mirror observations in the clinical setting; for example, IL-6, CXCL8 and TNF-α are elevated following COPD exacerbation in induced sputum or bronchoalveolar lavage samples ^15– 17^. The data presented here also agree with previous work assessing cytokine responses in a less extensively characterised human lung tissue model, in which TNF-α, IL-1β, IL-6, CXCL8 and IL-10 production was observed following LPS exposure or following influenza virus induced inflammation ^18, 19^.

IL-1β stimulation resulted in an induction of mediators where only 7 of the analytes measured increased by significant levels, and the magnitude of effect was lower than that seen with LPS stimulation, reflecting the more selective induction of signalling pathways with this agonist.

There is a pressing need for the development of new human disease models, in particular those which help reduce the need for animal models ^2, 8, 20^. There are intensive efforts to reconstitute the key components of the airway to generate clinically relevant *in vitro* models to be used in basic research and compound evaluation, including lung-on-a chip ^21^, dendritic cell-epithelium-fibroblast scaffolds ^22^ and differentiated epithelial cell layers ^23^. These approaches have both strengths and weaknesses; applicability to scale up is a strength but none are fully representative of an *in vivo* human lung. One of the advantages of the lung explant model over models such as air liquid interface culture of epithelial cells is maintenance of *in vivo* cell architecture without the need to induce differentiation in culture. Another approach that is growing in popularity and shares many of the advantages of an *ex vivo* tissue model is precision cut lung slices which provide a scaled model of the explant approach ^24, 25^. However, preparing precision cut lung slices from human tissue is technically much more difficult than from mouse tissue, and for the study of inflammatory approaches (as opposed to contractile responses) it offers no real advantages.

It is also possible that the use of a human tissue based approach could reduce the use of animals in target validation and overcome some of the obstacles and pitfalls faced when progressing from pre-clinical studies with animals to human trials ^2^. However, it is also important to note that there are limitations; including limited accessibility to human tissue, the relatively heterogeneous nature of resection samples, and natural donor variation in responses.

LPS raw data Luminex raw data for LPS stimulated tissue (Donors n=6). Click here for additional data file. Copyright: © 2017 Rimington TL et al. 2017 Data associated with the article are available under the terms of the Creative Commons Zero "No rights reserved" data waiver (CC0 1.0 Public domain dedication).

IL-1b raw data Luminex raw data for IL-1b stimulated tissue (Donors n=4). Click here for additional data file. Copyright: © 2017 Rimington TL et al. 2017 Data associated with the article are available under the terms of the Creative Commons Zero "No rights reserved" data waiver (CC0 1.0 Public domain dedication).

## Conclusions

In summary, we have demonstrated proof of concept that an *ex vivo* human lung tissue explant model can be used to mimic airway inflammation and provide low/medium throughput screening of anti-inflammatory properties of candidate drugs for the treatment of airway disease. This model should also help with target validation and reduce the reliance on animal models, thus reducing animal usage in the drug development process.

## Data availability

The data referenced by this article are under copyright with the following copyright statement: Copyright: © 2017 Rimington TL et al.

Data associated with the article are available under the terms of the Creative Commons Zero "No rights reserved" data waiver (CC0 1.0 Public domain dedication). 

**Dataset 1: LPS raw data.** Luminex raw data for LPS stimulated tissue (Donors n=6).

DOI, 10.5256/f1000research.10961.d157792 ^26^

**Dataset 2: IL-1b raw data.** Luminex raw data for IL-1b stimulated tissue (Donors n=4).

DOI, 10.5256/f1000research.10961.d157794 ^27^

## Supplementary material

**Supplementary Table 1: Patient demographics.**

**Supplementary Table 2: Custom Luminex panel design and standard curve range of analytes measured.**

**Supplementary Table 3: Concentration of analytes determined by Luminex.**
