# Supplementary material for: Defining the inflammatory signature of human lung explant tissue in the presence and absence of glucocorticoid
Source: F1000Res. 2017 Apr 11;6:460. [Version 1] doi: 10.12688/f1000research.10961.1 (PMC5497818; doi:10.12688/f1000research.10961.1)
Supplement: Supplementary file 3 [file f1000research-6-11814-s0002.tgz › 1c0bc79d-cd69-4836-bc6a-71c9d39a74fa.pdf]

Supplementary Table 1: Patient demographics.

Donor ID	D1	D2	D3	D4	D5	D6
**Age**	73	70	65	84	86	56
**Gender**	Male	Female	Female	Male	Female	Female
**FEV1 (% predicted)**	unknown	50.6	79.0	63.0	99	93.0
**FEV1/FVC (%)**	unknown	52.0	54.0	61.5	79.7	87.5
**Smoking status**	Ex-smoker	Recent smoker	Recent smoker	Ex-smoker	Ex-smoker	Never smoker
