# Supplementary material for: Defining the inflammatory signature of human lung explant tissue in the presence and absence of glucocorticoid
Source: F1000Res. 2017 Apr 11;6:460. [Version 1] doi: 10.12688/f1000research.10961.1 (PMC5497818; doi:10.12688/f1000research.10961.1)
Supplement: Supplementary file 4 [file f1000research-6-11814-s0003.tgz › 21246eae-7178-4247-95b8-387091854fb2.pdf]

Supplementary Table 2: Custom Luminex panel design and standard curve range of analytes measured.

Analyte	Standard curve range (pg/mL)
IL-8/CXCL8	17.7-7,310
MIP-1α/CCL3	314-25,440
MIP-1β/CCL4	145-11,720
MDC/CCL22	58.0-14,100
GM-CSF	27.2-6,610
VEGF	8.12-1,970
IL-4	16.9-4,100
IL-6	20.1-4,880
IL-10	14.8-3,600
TNF- α	19.8-4,800
IL-1β	10.0-2,430
IL-2	28.5-6,920
IL-17A	18.6-4,530
MCP-4/CCL13	6.17-1,500
MCP-1/CCL2	43.5-3,520
IL-12 p70	249-60,540
Eotaxin/CCL11	123-29,800
Eotaxin-3/CCL26	27.7-6,740
IFN-γ	18.9-1,530
IL-5	15.8-3,830
TARC/CCL17	94.2-22,900
