# Supplementary material for: Defining the inflammatory signature of human lung explant tissue in the presence and absence of glucocorticoid
Source: F1000Res. 2017 Apr 11;6:460. [Version 1] doi: 10.12688/f1000research.10961.1 (PMC5497818; doi:10.12688/f1000research.10961.1)
Supplement: Supplementary file 5 [file f1000research-6-11814-s0004.tgz › 591b78f2-7e21-4d4c-bb11-92137efcd881.pdf]

Supplementary Table 3: Concentration of analytes determined by Luminex.

Luminex assays were performed on cell culture supernatants from ex-vivo lung tissue explants, cultured under inflammatory conditions using LPS and IL-1β with or without dexamethasone. Analyte concentrations are normalised to account for wet tissue mass and presented as mean with the range provided in brackets. The number of donors are n=6 for LPS stimulations and n=4 for IL-1β stimulations. Of the 21 analytes measured, 12 generated quantifiable signals (the data was within the range of the standard curves for the assay) at the dilutions tested (either neat or 1 in 500). Following pro-inflammatory stimulation, levels of the majority of these analytes were increased. This inflammatory response was attenuated with dexamethasone treatment.

**Chemokines**						
	**CXCL8**	**CCL3**	**CCL4**	**CCL22**	**GM-CSF**	**VEGF**
**Conc basal (pg/mL/mg)**	2947.3 (282.7-7926.6)	22.7 (7.0-71.7)	4.5 (0.0-13.8)	11.1 (2.8-37.1)	1.7 (0.0-5.8)	25.1 (18.8-33.5)
**Conc LPS (pg/mL/mg)**	10211.0 (724.1-17356.1)	816.8 (188.5-1557.7)	79.6 (5.9-204.0)	14.0 (6.0-33.8)	37.9 (25.0-56.0)	47.3 (31.4-68.1)
**Conc LPS+Dex (pg/mL/mg)**	4843.1 (250.1-8989.4)	297.2 (84.6-718.9)	35.8 (4.4-118.5)	10.9 (5.4-21.4)	16.9 (8.8-32.7)	14.0 (4.6-24.3)
**Conc IL-1β (pg/mL/mg)**	5780.9 (615.6-11737.0)	76.3 (35.3-102.4)	7.1 (1.6-13.4)	9.8 (1.8-20.8)	29.6 (23.0-45.0)	21.7 (13.3-33.2)
**Conc IL-1β+Dex (pg/mL/mg)**	2897.7 (486.8-5069.2)	28.8 (22.5-35.6)	4.0 (1.7-7.8)	11.2 (3.3-26.9)	15.6 (13.8-17.4)	7.8 (4.9-10.3)
**Cytokines and other factors**						
	**IL-4**	**IL-6**	**IL-10**	**TNF- α**	**IL-1β**	**IL-2**
Conc basal (pg/mL/mg)	0.6 (0.4-0.9)	891.4 (129.8-1794.8)	0.1 (0.0-0.6)	0.0 (0.0-0.1)	1.5 (0.0-4.3)	4.2 (2.5-6.2)

Conc LPS (pg/mL/mg)	1.1 (0.7-1.7)	3511.8 (348.4-5808.0)	5.5 (3.7-9.8)	29.9 (10.3-54.5)	54.0 (20.2-138.0)	5.6 (3.3-9.2)
Conc LPS+Dex (pg/mL/mg)	0.8 (0.4-1.1)	881.9 (62.8-2628.4)	2.6 (1.4-6.2)	10.2 (2.6-43.1)	24.1 (6.0-83.8)	6.3 (3.7-9.4)
Conc IL-1β (pg/mL/mg)	0.6 (0.4-1.0)	2010.4 (165.1-4523.4)	1.3 (0.9-1.6)	0.7 (0.3-1.3)	n/a	3.7 (2.4-5.2)
Conc IL-1β+Dex (pg/mL/mg)	0.5 (0.3-0.7)	565.8 (75.3-1225.3)	0.9 (0.5-1.1)	0.2 (0.0-0.5)	n/a	3.8 (2.9-5.4)
