# Supplementary material for: Defining the inflammatory signature of human lung explant tissue in the presence and absence of glucocorticoid
Source: F1000Res. 2017 Apr 11;6:460. [Version 1] doi: 10.12688/f1000research.10961.1 (PMC5497818; doi:10.12688/f1000research.10961.1)
Supplement: Peer review history

# Peer review: Referee response for version 1

**DOI:** 10.5256/f1000research.11814.r21789

**Published:** 2017-06-27

**Authors:** Colin D. Bingle

**Affiliations:** 1 Academic Unit of Respiratory Medicine, Department of Infection, Immunity & Cardiovascular Disease, University of Sheffield, Sheffield, UK

Tracy Rimington and colleagues present a novel preliminary analysis of the inflammatory signature of human lung explant tissue. They correctly identify that limitations in human lung tissue models has hindered pre-clinical and mechanistic studies and provide data to suggest that a simple explanted lung tissue model may have some utility in this area.

 A significant advantage of this model is the lack of requirement for complex model development and should allow the technique to be utilised in any tissue culture facility with ease. The model takes multiple small pieces of human lung tissue and cultures them in a standard growth media for periods of up to 24 hours. Media is removed and then can be used for downstream assays. In this case media was used in a custom Luminex assay to detect a range of inflammatory mediators.

 The results suggest that the model allows for the detection if secretion of 12/21 analytes chosen and shows that secretion of these was modified by inclusion of the pro-inflammatory mediators, bacterial LPS and interleukin beta into the media.

 This preliminary data suggests that this type of model could be further developed and may become a valuable tool for pulmonary research.

 The current data shows how there is a high level of variability between different explants, both in terms of basal and stimulated levels. The reasons for this will need to be explored. Does it represent true variability between different donors or could it be due to the way in which individual donor explants are generated? Maybe the size of each tissue fragment will have a significant influence on responses?

 It will also be helpful to explore the tissue viability and to investigate extending the time frame of the explant cultures? Again perhaps tissue fragment size will be an important variable.

 Overall, the data provide a useful primer to the further development of lung tissue explants as a research tool.

I have read this submission. I believe that I have an appropriate level of expertise to confirm that it is of an acceptable scientific standard.

# Peer review: Referee response for version 1

**DOI:** 10.5256/f1000research.11814.r21786

**Published:** 2017-04-26

**Authors:** Yassine Amrani

**Affiliations:** 1 Department of Infection, Immunity and Inflammation, University of Leicester, Leicester, UK

This interesting report describes the use of human lung tissue explants as an *in vitro* model to study responses to various proinflammatory stimuli. The authors showed that the model can be cultured for 24 hr and retains responsiveness to LPS and IL-1β with the production of different cytokines and growth factors. Interestingly, the proinflammatory potential of cultured human lung tissue explants can still be modulated by dexamethasone, suggesting that the model would be ideal to test novel anti-inflammatory drugs. Overall the manuscript is clearly written and data are novel. There are some minor points that need clarifications.

  

1. It would important to state how tissue viability was assessed following the 2 days culture
2. Please also provide, if possible, some representative picture of the histology of human lung tissue explants before and following the 2 day culture
3. It would better to express the data in Figure 1 as net cytokine increase rather than % of LPS. 

I have read this submission. I believe that I have an appropriate level of expertise to confirm that it is of an acceptable scientific standard.
